# Tracking hatchery and wild Atlantic salmon (*Salmo salar*) smolt during river exit and early marine migration to assess survival, predation risk and migration speed

**DOI:** 10.1111/jfb.70599

**Published:** 2026-07-30

**Authors:** Natalie C. Koopman, David C. Hardie, Robert J. Lennox, Morgan L. Piczak, Frederick G. Whoriskey, Eric B. Brunsdon, Jeffrey A. Hutchings, Glenn T. Crossin

**Affiliations:** ^1^ Biology, Dalhousie University, Department of Biology Life Science Centre, Dalhousie University Halifax Nova Scotia Canada; ^2^ Fisheries and Oceans Canada Dartmouth Nova Scotia Canada; ^3^ Ocean Tracking Network, Dalhousie University Halifax Nova Scotia Canada

**Keywords:** Atlantic salmon, hatchery, predation, smolt, survival, telemetry

## Abstract

Significant declines in Atlantic salmon (*Salmo salar*) populations across their range have prompted an increased use of hatchery supplementation programmes to support population recovery. However, concerns persist regarding the effectiveness of these programmes due to limited understanding of how hatchery‐reared salmon perform post‐release in natural river systems compared to their wild counterparts. This study examined survival, predation risk, migration speed, river exit timing and early ocean migration patterns of hatchery‐reared and wild Atlantic salmon smolts in the LaHave River, Nova Scotia, where populations are among the most dramatically reduced in Canada. Using acoustic telemetry, we tracked the movements of 100 smolts (50 hatchery origin, 50 wild origin) during their freshwater downstream migration and into the ocean. Compared to wild smolts, hatchery smolts exhibited significantly lower survival during river outmigration, with a third dying before reaching lower river sections. Predation was a major driver of mortality, with hatchery smolts experiencing over four times higher predation risk than wild smolts following release. Hatchery smolts had significantly slower migration speeds in the upper river and lake sections, and they also exhibited significantly greater reverse movements near the head of tide compared to wild smolts. However, river exit timing and early ocean migration routes were comparable between smolts' origins, indicating potential synchrony and environmental cue retention for hatchery smolts. Critical empirical evidence of early marine migration for the salmon population was captured, with both hatchery and wild smolts following the expected eastward migration along the southern coast of Nova Scotia. Taken together, these results indicate that hatchery smolts experience higher predation risk and less efficient migration than wild smolts, likely due to predator naivety and captive rearing conditions. This study also identified a predation hotspot in Wentzells Lake, the one lake smolts migrate through during river exit, highlighting an opportunity to increase smolt survival by releasing hatchery smolts downstream of high‐risk predation areas. Implementing targeted release strategies and innovative captive rearing conditions may help mitigate post‐release challenges for hatchery smolts.

## 1 INTRODUCTION

Steep declines in Atlantic salmon (*Salmo salar*; from herein salmon) populations have been documented across much of their native range over the past several decades (Fraser, 2016; ICES, 2022; Pardo et al., 2021). Increasing stressors within freshwater habitats continue to play a significant role in driving these declines (Gillis et al., 2023; Todd et al., 2011). Warmer water temperatures (Gillis et al., 2023), habitat loss, acidification, migration barriers (Forseth et al., 2017; ICES, 2022; Thorstad et al., 2021) and the spread of invasive species (Bean, 2020; Kennedy et al., 2018) are all documented threats for salmon during their freshwater life stages. Within Atlantic Canada, several salmon populations have experienced severe declines, particularly those in the Nova Scotia Southern Uplands Designatable Unit (NSSU DU; Raab et al., 2024). In 2010, populations in the NSSU DU were assessed as Endangered by the Committee on the Status of Endangered Wildlife in Canada (COSEWIC, 2010).

In response to declining populations, hatchery programmes have been frequently used as a management tool to restore fish populations, including salmon (Leber et al., 2005; McMillan et al., 2023). Hatcheries typically capture returning adult salmon as broodstock, spawn the adults in captivity, then rear their offspring in captivity until releasing the juveniles back into the river at some juvenile stage (egg, fry, parr or smolt) to increase recruitment (Fraser, 2016; Lennox et al., 2021). Hatcheries have played a large role in salmonid management for over 150 years, serving economical (commercial fisheries support), recreational (stocking for more recreational angling opportunities) and conservation purposes (population recovery and supplementation; Bordeleau et al., 2018; Maynard & Trial, 2014). In theory, hatcheries aim to reduce juvenile mortality and boost population productivity. In the wild, fewer than 2% of salmon typically survive from egg to smolt (Cunjak & Therrien, 1998). By providing a controlled environment with a reliable feeding regime and predator protection, hatcheries greatly improve early survival rates, allowing more juveniles to be released than would naturally occur.

Despite hatcheries' intended benefits, it is well‐documented that captive conditions deprive fish of natural experiences and environmental cues, resulting in altered behaviour and physiology compared to their wild counterparts (Araki et al., 2008; Fleming & Gross, 1993; Fleming & Petersson, 2001; Fraser, 2008; Jonsson, 1997). Observed differences between hatchery and wild salmon may arise through the genetic and domestication selection of favouring smolt characteristics better suited to captivity over successive generations (Araki et al., 2008; Milot et al., 2013), and also from the altered environment in hatcheries that can limit early‐life development and experiences (Johnsson et al., 1996; Olla et al., 1998). For example, wild smolts in natural river systems are exposed to environmental complexity, predation risk and variable resource availability, shaping behavioural traits such as foraging efficiency, predator recognition and competitive interactions (Einum & Fleming, 1997; Järvi & Uglem, 1993; Jonsson et al., 2019; Sabal et al., 2020; Vilhunen, 2006). In contrast, hatchery‐reared juveniles experience simplified, low‐risk conditions with minimal habitat complexity, no predation pressure and reduced variation in size structure. Under these conditions, behaviours such as aggression and boldness may be favoured, as the costs of risk‐taking are low relative to those in the wild (Fleming & Einum, 1997; Huntingford, 2004; Jonsson et al., 2019). Evidence from both observational and experimental studies indicates that hatchery conditions during early life stages can have lasting, detrimental effects on behaviour and performance, including differences in aggression, risk‐taking and reproductive success between hatchery and wild fish (Fleming et al., 1997; Jonsson et al., 2019). Consequently, hatchery‐reared fish may enter the smolt stage with behavioural and physiological traits that are poorly suited for survival and migration in natural environments. These outcomes raise concerns about the effectiveness of hatchery programmes, especially if released individuals are not behaviourally and physiologically similar to the wild population, and are therefore not as adept at survival and reproduction in the natural environment (Brown & Day, 2002; Carr et al., 2004; Salvanes & Braithwaite, 2006). Lennox et al. (2023) conducted a review of 19 supplementation studies and found only two programmes reported success in increasing fish numbers and creating a self‐sustaining population. Efforts such as predator conditioning and environmental enrichment have been proposed as management solutions (Janhunen et al., 2021; Vilhunen, 2006), yet the long‐term success of these interventions remains uncertain. As a result, many conservation hatchery programmes continue to face criticism for their limited success in reversing population declines (Araki et al., 2008; Fraser, 2008) and their efficacy when used as a long‐term recovery strategy (Almodóvar et al., 2020; Christie et al., 2012; Young et al., 2017).

The salmon population within the LaHave River has been monitored continuously since 1970, providing a long‐term record that is critical for establishing baseline conditions and informing management responses to the drastic decreases in salmon abundance (Raab et al., 2024; Gibson et al., 2011). Despite decades of monitoring and extensive hatchery stocking, the population continues to decline. Over the last three generations, adult returns have decreased by 83%, while juvenile densities of all life stages have dropped by 73%–87% (Raab et al., 2024). Since the 1970s, millions of hatchery‐reared juveniles have been introduced into the system in an effort to bolster recruitment (Raab et al., 2024). However, despite sustained stocking efforts, smolt‐to‐adult return rates have remained critically low (below 1%) following the last major smolt stocking events of ~700,000 fry, 274 smolt and 385 adults being released from 2017 to 2020 (Raab et al., 2024). Compounding the challenges already faced by smolts in this system, invasive aquatic predators such as smallmouth bass (*Micropterus dolomieu*) and chain pickerel (*Esox niger*) have been identified as predators of salmon smolts within the LaHave River (Feener, 2017). Given that predation is a major potential source of freshwater mortality during outmigration, these invasive species may represent an important selective pressure on smolt survival (Mensinger et al., 2025). Predation risk is often driven by behavioural traits such as boldness, vigilance and habitat use, which may differ between hatchery and wild smolts as a result of their contrasting rearing environments. However, it remains unclear whether such behavioural differences exist during outmigration for LaHave River smolts and, if so, whether they result in different predation rates between origins. Understanding whether hatchery smolts are disproportionately predated upon compared to wild smolts is therefore essential to evaluating the effectiveness of supplementation efforts.

This study examines how survival, predation, river movement, river exit timing and ocean migration differ between wild and hatchery‐reared Atlantic salmon smolts during their seaward outmigration in the LaHave River, Nova Scotia. We hypothesized that hatchery‐reared smolts would experience lower survival rates, higher predation risk, exhibit less efficient outmigration, including slower migration speeds and delayed river exit, and deviate from the typical oceanic migration pathways compared to their wild counterparts. We propose that these differences between hatchery and wild smolts reflect carryover effects of captivity, arising from the markedly altered conditions and experiences that shape early development in hatchery settings compared to natural river systems. Using acoustic transmitters equipped with temperature and predation sensors, we tracked the downstream migration of wild and hatchery‐reared smolts through the LaHave River and estuary, and into the Atlantic Ocean. Our findings offer key insights into the challenges faced by hatchery‐reared smolts upon release into the wild, and inform future best practices for supplementation programmes that intend to restore salmon populations.

## 2 METHODS

### 2.1 Study site

Salmon smolts were tagged on the LaHave River, which is located on the southern shore of Nova Scotia within the NSSU DU (Figure 1). The headwaters originate in Annapolis County and flow 97 km before draining into the Atlantic Ocean, with a total drainage area of 1670 km^2^ (Gray et al., 1989). Wentzells Lake, located 36 km upstream from the river mouth and 10 km downstream of Morgan Falls, is the only lake in the LaHave River system that smolts in this study would have encountered during their migration (Figure 1). The lake is approximately 2 km long and 1 km wide, with shoreline housing and recreational boating activity.

**FIGURE 1:**
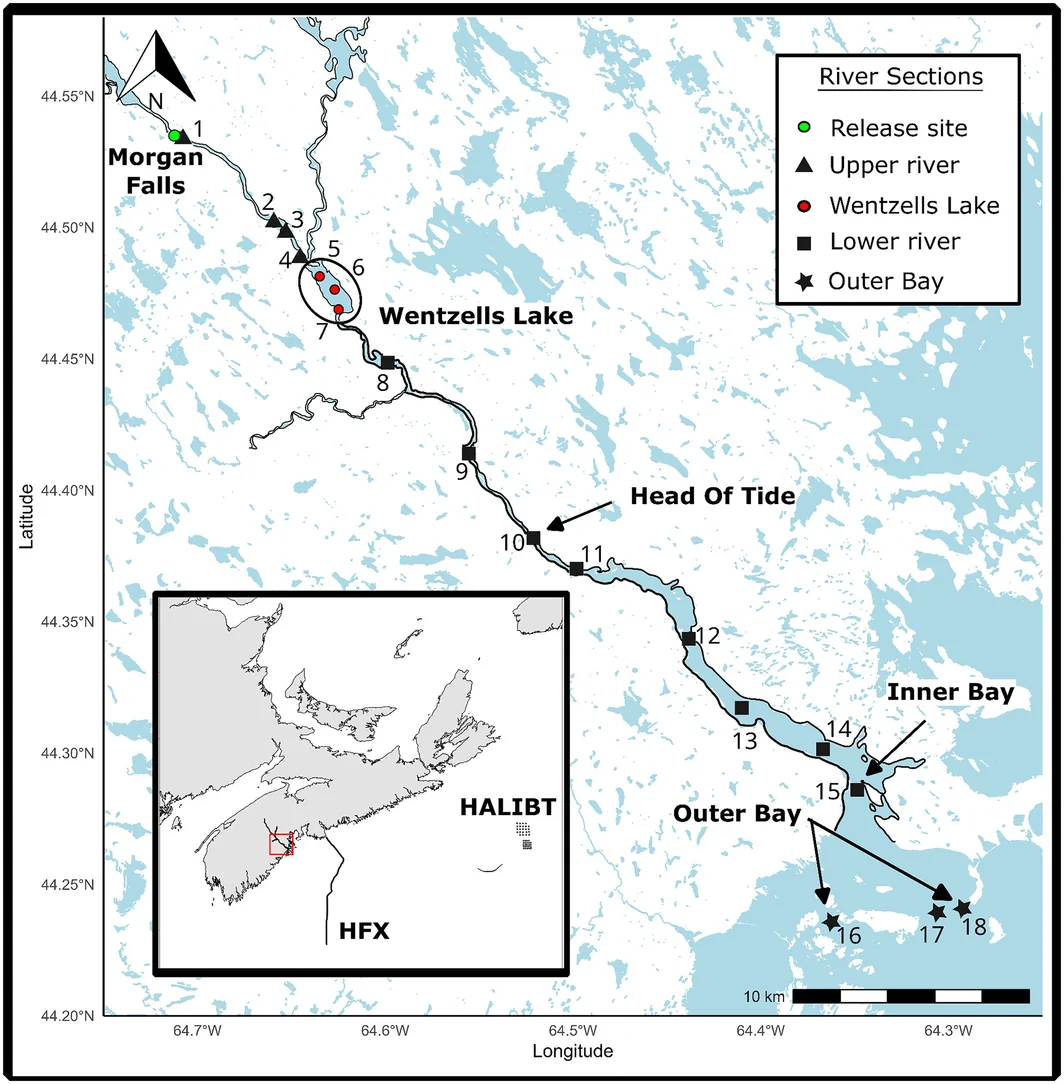
Map of the LaHave River acoustic receiver deployment locations for April–July 2021 and defined river sections. Important river locations, such as Morgan Falls, Wentzells Lake, the head of tide, inner bay exit receiver and the outer bay array, are labelled. The inset map with a red box indicates the location of the LaHave River in Nova Scotia, Canada. The marine acoustic arrays of the Halifax Line (HFX) and the HaliBT project (HALIBT) are represented and labelled.

Adult salmon returns and juvenile densities have substantially declined in the past few decades for the LaHave River salmon population (Bowlby et al., 2013), with modelling revealing a high probability of the population becoming extirpated in the next 50 years if there are no changes in current survival rates (Raab et al., 2024). Although stocking on the LaHave River likely began in the 1800s, hatchery releases and salmon population monitoring have only been systematically recorded since the 1970s via electrofishing, mark‐recapture studies and fish ladder observations (Bowlby et al., 2013; Raab et al., 2024). The majority of monitoring has taken place upstream of Morgan Falls, a hydroelectric power station located 45 km from the river mouth and equipped with a fish ladder. Fifty‐one percentage of the river's accessible habitat lies above Morgan Falls, with reports of estimated egg deposition not reaching the conservation requirement since the fishway was opened in the 1970s (Raab et al., 2024). Captive‐reared juveniles have been released above and below Morgan Falls from the 1970s to 2005, with a break in the stocking until 2016. One of the most recent releases occurred in 2020, where 270,000 fry, several hundred smolts and 93 adults of native broodstock origin were released into the LaHave River (Raab et al., 2024). Record high historic escapement counts of 1SW salmon occurred in 1988, with 3520 individuals who spent 1 year at sea (1SW) and 449 adults spent at least 2 years at sea (MSW) recorded at Morgan Falls fish ladder (Bowlby et al., 2013). In 2021, a total of 176 returning individual adult salmon were recorded, including 157 1SW and 19 MSW. Thus, despite efforts of over 3.2 million hatchery juveniles being released in the LaHave River over time, adult returns continue to decline with return rates falling to 0.5%, suggesting little success of population recovery (Bowlby et al., 2013).

### 2.2 Receiver deployment

Salmon were tracked using acoustic receivers deployed strategically in the LaHave River system (Figure 1). A total of 18 Innovasea VR2W‐69 kHz receivers (Innovasea, Bedford, Nova Scotia, Canada) were deployed throughout the freshwater, estuary and bay of the LaHave River in April 2021 to track the outmigration of tagged smolts (Figure 1). The receivers were categorized into river sections to better understand smolt behaviour and movement in each area of the river. Following release at Morgan Falls, smolts initially enter the Upper River before progressing into Wentzells Lake. After traversing the lake, individuals move through the Lower River and subsequently enter the Outer Bay, marking the transition into the marine environment. The receivers were removed and data downloaded by July 2021.

Detection data from marine receiver arrays were sourced via the Ocean Tracking Network (OTN), which compiles acoustic telemetry data from coordinated deployments across the Northwest Atlantic. Each array represents a spatially defined cluster of passive acoustic receivers positioned throughout the ocean to detect tagged animals as they travel through these monitored regions.

### 2.3 Experimental design

#### 2.3.1 Study species

Salmon of both wild origin from the LaHave River and hatchery‐reared were used in this study. Salmon of hatchery origin were offspring from adult LaHave River broodstock salmon spawned in the fall of 2018 and reared at the Coldbrook Biodiversity Facility, Nova Scotia. Once the hatchery juveniles reached the smolt stage in the spring of 2021, they were transported to Morgan Falls in aerated tanks and placed in circular holding tanks supplied with water from the LaHave River. Smolts were fed every 3 days before tagging. Wild‐origin smolts were captured at the Morgan Falls power station fish trap, which was checked three times a week. Captured wild smolts were held in individual flow‐through crates with water coming from the LaHave River and were separated by day of capture. The term ‘wild‐origin’ smolts is used to acknowledge that, although the fish were captured in the wild, the river has been subject to prolonged stocking, and the population may therefore not represent a wholly wild lineage.

#### 2.3.2 Tagging procedure

A total of 100 smolts, 50 wild and 50 hatchery, were tagged at a 1:1 ratio each day of tagging at the Morgan Falls power station (Table 1). Tagging occurred in the spring of 2021 with Innovasea 69 KHz V7DT acoustic predation tags (7 mm X 22 mm, 1.7 g in air, R64k protocol, 27 days of battery; Table 1). The V7DT tags provide a direct measurement of predation wherein the predator's stomach acids digest a tag's polymer‐coated sensor, altering the signal of the tag to indicate the fish has been eaten and triggering a timer used to determine when the predation event occurred. Tags were also equipped with a temperature sensor ranging from 0 to 40°C to provide further information on what type of predator digested the smolts. Endotherms, birds (with body temperatures around 40°C) and mammals (ranging from 36 to 39.5°C) maintain significantly higher and more distinguishable body temperatures compared to aquatic, poikilothermic predators whose body temperatures closely match the surrounding water. The temperature that is measured at the time of predation is then transmitted for the rest of the tag's life (Figure S1).

**TABLE 1:** Number of smolts tagged and released on the LaHave River by release day, origin, means and standard deviation for the fork length and weight (range in parentheses).

Release day	Origin	*n*	Length (cm)	Weight (g)
			Mean SD range	Mean SD range
29 April 2021	Hatchery	6	21.5 ± 1.9 (19–24.5)	95.3 ± 23.6 (63.8–128)
	Wild	6	19.9 ± 1.0 (18.6–21.2)	75.0 ± 13.0 (58.7–93)
1 May 2021	Hatchery	8	21.8 ± 1.5 (18.9–23.9)	101 ± 17.3 (72.8–133)
	Wild	8	22.0 ± 2.2 (22.0–26.5)	104 ± 35.2 (73.7–174)
3 May 2021	Hatchery	11	21.1 ± 2.3 (17.4–24)	90.4 ± 26.1 (55.4–123)
	Wild	11	20.1 ± 1.5 (17.9–22.8)	77.8 ± 18.1 (51.3–108)
5 May 2021	Hatchery	11	21.7 ± 2.2 (17.6–24.8)	95.3 ± 30.9 (47–150)
	Wild	11	20.7 ± 2.6 (15.6–24.4)	87.8 ± 27.0 (42.5–129)
7 May 2021	Hatchery	8	21.0 ± 1.2 (19.3–22.4)	89.9 ± 14.9 (65.4–108)
	Wild	8	19.4 ± 1.4 (17.4–21.3)	74.1 ± 20.1 (49.1–111)
9 May 2021	Hatchery	6	21.7 ± 1.0 (20.7–23.2)	92.9 ± 12.0 (80.3–112)
	Wild	6	21.6 ± 0.9 (20–22.7)	99.1 ± 10.2 (92–120)
Total	Hatchery	50	21.4 ± 1.8 (17.4–24.8)	93.8 ± 22.8 (47.0–149.7)
	Wild	50	20.6 ± 2.0 (15.6–26.5)	85.8 ± 25.1 (42.5–174.2)

Surgical tools and tags were disinfected in Betadine and rinsed in saline solution before each surgery. Smolts were anaesthetized in an aerated bucket with tricaine methanesulfonate (TMS) until loss of equilibrium. Weight and fork length were taken before the fish was placed ventral side up on a moist sponge, where gills were constantly irrigated with a maintenance dose of TMS. A small incision was then made on the mid‐ventral line, and the sterile acoustic transmitter was placed intraperitoneally. The incision was closed with two interrupted sutures. The smolts were immediately placed in a recovery holding tank until they regained equilibrium and swimming capacity. Once recovered, they were transferred to a flow‐through crate, where all tagged smolts were held for a minimum of 5 h before being released in the LaHave River later that same day.

### 2.4 Data analysis

All data were processed and figures produced using R (Version 4.3.3; R Core Team, 2024).

Raw acoustic detections were processed using the actel package function *migration* (Flávio & Baktoft, 2021). Individual detection plots were examined for atypical movement patterns, such as extended periods of non‐detection. To ensure that no false detections or implausible movements were included, Actel triggered warnings that were manually reviewed when smolts skipped two or more receivers or when smolt speeds between receivers exceeded 3 m/s [which is a conservative threshold reflecting biologically plausible swim speeds (Remen et al., 2016), given the low flow rate of LaHave River (~1.01 m/s; COSEWIC, 2010)]. Fish movements immediately before and after predation tag activation were also reviewed to ensure that no post‐predation detections were included in the analysis. To exclude post‐predation detections, data were filtered based on predation tag sensor values. The predation sensor remains at 1 under normal conditions but increases (>1) once the tag is ingested, representing the time since digestion.

#### 2.4.1 Survival and predation

To determine whether hatchery rearing affected survival, we used a Cormack–Jolly–Seber (CJS) model (Cormack, 1964; Jolly, 1965; Seber, 1965) using the RMark package (Laake, 2013). The CJS model is commonly used in mark‐recapture studies to estimate the key parameters of apparent survival (ϕ), defined as the probability that a fish is alive and remains in the study area between detections, and detection probability (p), defined as the probability a fish is detected if it is alive. The model uses a maximum likelihood method, determining the survival and detection values that best explain the observed data. Each fish's detection history was represented as a binary matrix, indicating presence (1) or absence (0) at each receiver location over time. Given the assumed downstream, unidirectional outmigration of smolts, each survival interval corresponded to the section between two adjacent receiver arrays.

Fish were considered to have survived outmigration if they were detected at the inner bay receiver (receiver 15, Figure 1) or any subsequent outer bay receivers. Individuals were also classified as mortalities if their predation tag sensor was triggered. For fish not exhibiting predation signals, fate was inferred based on their final detections. Fish whose last detection occurred within the river and were subsequently not detected downstream were assumed to have died or emigrated from the study area. This assumption is supported by the high detection efficiency of the receiver arrays (calculated using the actel package; Flávio & Baktoft, 2021) and the expectation that healthy individuals continue to move downstream and remain detectable. A lack of downstream detections despite continued monitoring is considered a reliable indicator of in‐river mortality or tag failure (Klinard & Matley, 2020), although tag failure is unlikely given consistent detection histories prior to disappearance.

We built candidate models to test the influence of origin (hatchery vs. wild) and river section on apparent survival (ϕ). Detection probability (p) was modelled as a function of river section to account for spatial variation in detection (Table S2). Competing models included additive and interaction relationships between origin and river section, as well as a reduced model excluding origin [ϕ(river section), p(river section)] to determine whether including origin improved model support. Model support was assessed using Akaike's information criterion corrected (AICc) for small sample size, with lower AICc values and higher model weight indicating better support. Model selection strongly supported the additive model [ϕ(origin + river section), p(river section)], receiving the majority of model support (AICc = 559.53, ΔAICc = 0, model weight = 0.99998; Table S1). The model excluding origin was less supported, indicating that the inclusion of origin improved model fit and better explained smolt survival.

The CJS model addresses overall survival differences, but we expected that predation may be a large factor driving differences in survival between smolts of hatchery and wild origin. To assess the influence of origin on the risk of predation, we used a Cox proportional hazards model with time to predation event (in days since release) as the response variable via the *coxph()* function from the survival package (Therneau, 2024). Hazard (risk) of predation was modelled over time while including the explanatory variables origin (hatchery vs. wild), weight and release day as predictor variables.

#### 2.4.2 River movement and exit

For all movement analyses, we used the final detection at a site to indicate when a fish departed (e.g., began migrating) and the first detection to indicate arrival, corresponding to downstream movements. A smolt's river movement during outmigration is assumed to be largely unidirectional towards the ocean, with some potential reverse movement at the head of tide for saltwater acclimation (Halfyard et al., 2012; Kocik et al., 2009). Based on this assumption, we calculated individual migration speed through the four river sections (i.e., Upper River, Wentzells Lake, Lower River, Outer Bay) using the time elapsed between first detections at successive sections. To standardize for smolt size, migration speed was expressed in body lengths per second (bl/s). For each fish, detections were arranged chronologically, and the migration speed (m/s) was calculated by dividing the distance between detections (m) by the time elapsed between detections (s). The migration speed (in m/s) was then divided by the individual's body length (in m) to yield body lengths per second (bl/s). To compare the outmigration of hatchery and wild smolts, a generalized linear mixed‐effects model (GLMM) with a gamma distribution and log link was used, with migration speed (bl/s) as the response variable. Fixed effects include an interaction between the river section and origin, weight and release day. Fish ID was included as a random effect to account for repeated measures of migration speed across river sections for individual smolts (Table S4).

River exit timing was assessed by extracting detection histories using *detection_events* summarizing functions using the glatos package (Holbrook et al., 2024). Exit dates were converted to Julian day, and the duration of river residence for each individual was calculated as the difference in days between its river entry/release and exit date. River entry was considered the date on which they were released into the river. River exit was defined as the date of final detection at the inner bay receiver, which reflects the last confirmed presence in the estuarine section of the river system (Figure 1).

To assess whether hatchery rearing influenced fine‐scale migratory behaviour during downstream outmigration, we quantified the number of reverse (upstream) movements made by individual smolts in the lower river near the head of tide. Reverse movements were defined as movements in the upstream direction between successive receiver detections. Only detections from the lower river and bay receiver arrays were included to strictly assess tidal transitional zones and saltwater acclimation. Reverse movement counts were analysed using a generalized linear model (GLM) with a negative binomial error distribution to account for overdispersion in the count data. Origin (hatchery vs. wild), weight and release day were included as predictor variables.

Marine detection data from ocean arrays were obtained through OTN. To assess early marine migration, only detections occurring after river exit were retained, and 100% minimum convex polygons (MCPs) were generated around all ocean detections for hatchery and wild smolts. These MCPs illustrate the overall spatial extent of marine habitat use by each origin group.

## 3 RESULTS

A total of 100 salmon smolts (50 hatchery, 50 wild) were tagged and released in the LaHave River over a period of 6 days between 29 April and 9 May 2021 (Table 1). On each day, equal numbers of wild and hatchery smolts were released. Of those released, 98 individuals were detected. Two smolts (1 hatchery, 1 wild) were never detected and thus excluded from analyses. Among the 98 smolts, the glatos package aggregated 218,736 raw detections into 8364 movements events, from 29 April to 13 June 2021. Events were defined as either a change in the receiver location or a gap of more than 1 day between detections on the same receiver.

### 3.1 Survival and predation

Among the 98 smolts detected, 29% (*n* = 28) died during outmigration. Observed mortality differed by origin, with 41% (20 of 49) of hatchery smolts dying compared to 16% (8 of 49) of wild smolts. Model estimates from the CJS framework indicated higher cumulative survival in wild smolts (86.0%; 95% CI: 77.9–91.4%) compared to hatchery smolts (60.4%; 95% CI: 47.6%–72.0%; Table S3). The CJS additive model revealed that survival differences were concentrated in the Upper River and Wentzells Lake sections, with an estimated 39.6% (95% CI: 28.0%–52.4%) of hatchery smolts dying before reaching the lower river, compared to 14.0% (95% CI: 8.6%–22.1%) of wild smolts (Figure 2; Table S3). The rest of the river had comparable survival estimates between wild and hatchery smolts, demonstrating that the upper LaHave River and Wentzells Lake are areas that drive survival differences between hatchery and wild smolts (Figure 2).

**FIGURE 2:**
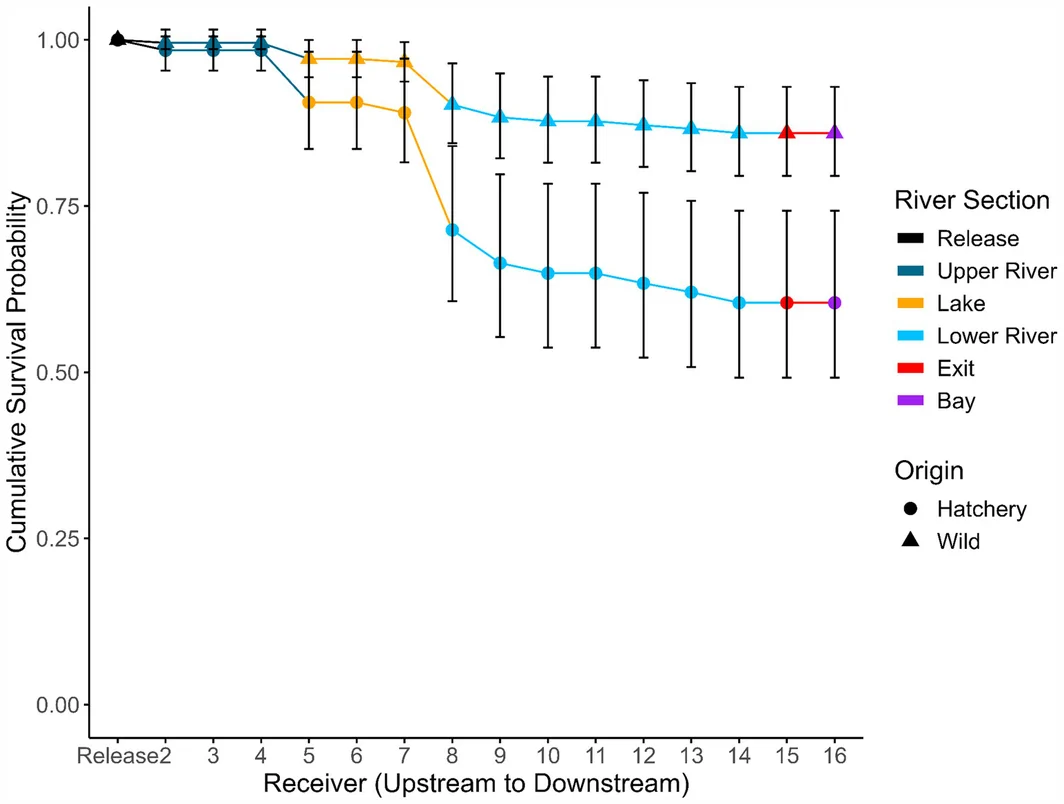
Estimated cumulative survival by river section for hatchery and wild Atlantic salmon smolts in the LaHave River, NS, based on a Cormack–Jolly–Seber survival model. Error bars represent the cumulative survival standard error.

Of the 20 hatchery smolts that died, 17 (85%) of these mortalities were identified as a predation event based on triggered digestion sensors. In contrast, eight wild smolts died, with four (50%) of these deaths determined as predation. All freshwater predation events occurred within the first 22 km downstream of the release site at Morgan Falls, with 11 events (52%) taking place in Wentzells Lake and an additional four occurring just upstream of the lake's entrance (Figure 3). The Cox proportional hazards model revealed that origin was a significant predictor of predation risk, with wild smolts exhibiting a hazard of predation that was 81% lower than that of hatchery smolts (*p* = 0.003). Neither weight nor release date was a significant predictor. Temperatures transmitted during all predation events were consistent with river temperatures reported for the LaHave River over May (7.5–16°C; Feener, 2017), supporting poikilothermic predation. No elevated temperatures were recorded that would indicate mammalian or avian predators.

**FIGURE 3:**
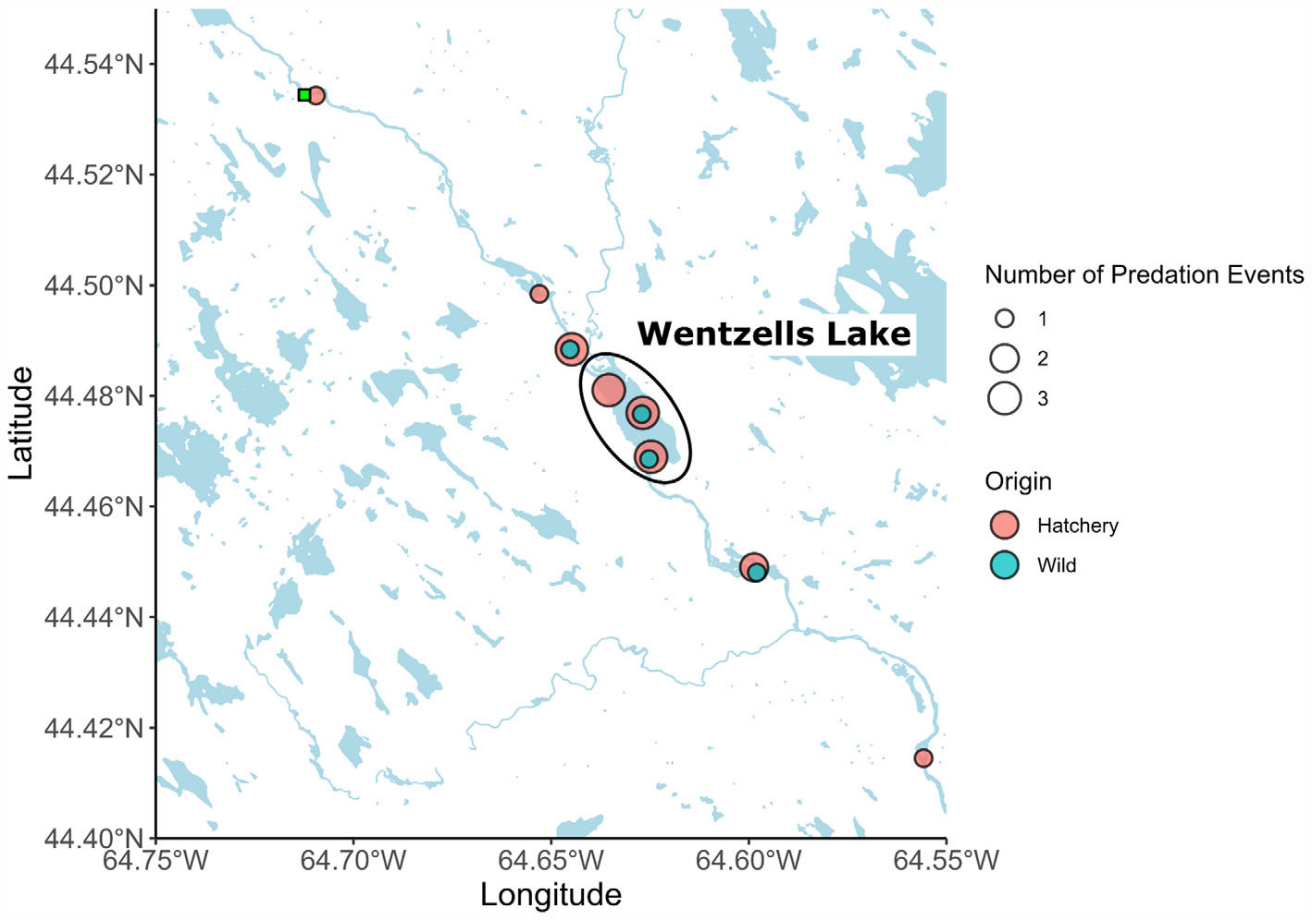
Map of predation events of hatchery and wild Atlantic salmon smolts on the LaHave River, with the size of a point representing the number of salmon predated at the location and the colour representing the origin of Atlantic salmon smolts (hatchery or wild).

### 3.2 River movement and exit

Migration speed differed across river sections. Relative to the Outer bay, smolts migrated faster in the lake (GLMM estimate = 2.12 bl/s ± 0.19 standard error (SE), *p* < 0.001) and Upper river (estimate = 2.07 bl/s ± 0.19 SE, *p* < 0.001). By contrast, migration speeds in the Lower river did not differ compared to migration speeds in the Outer bay (estimate = 0.02 bl/s ± 0.20 SE, *p* = 0.92; Figure 4a).

**FIGURE 4:**
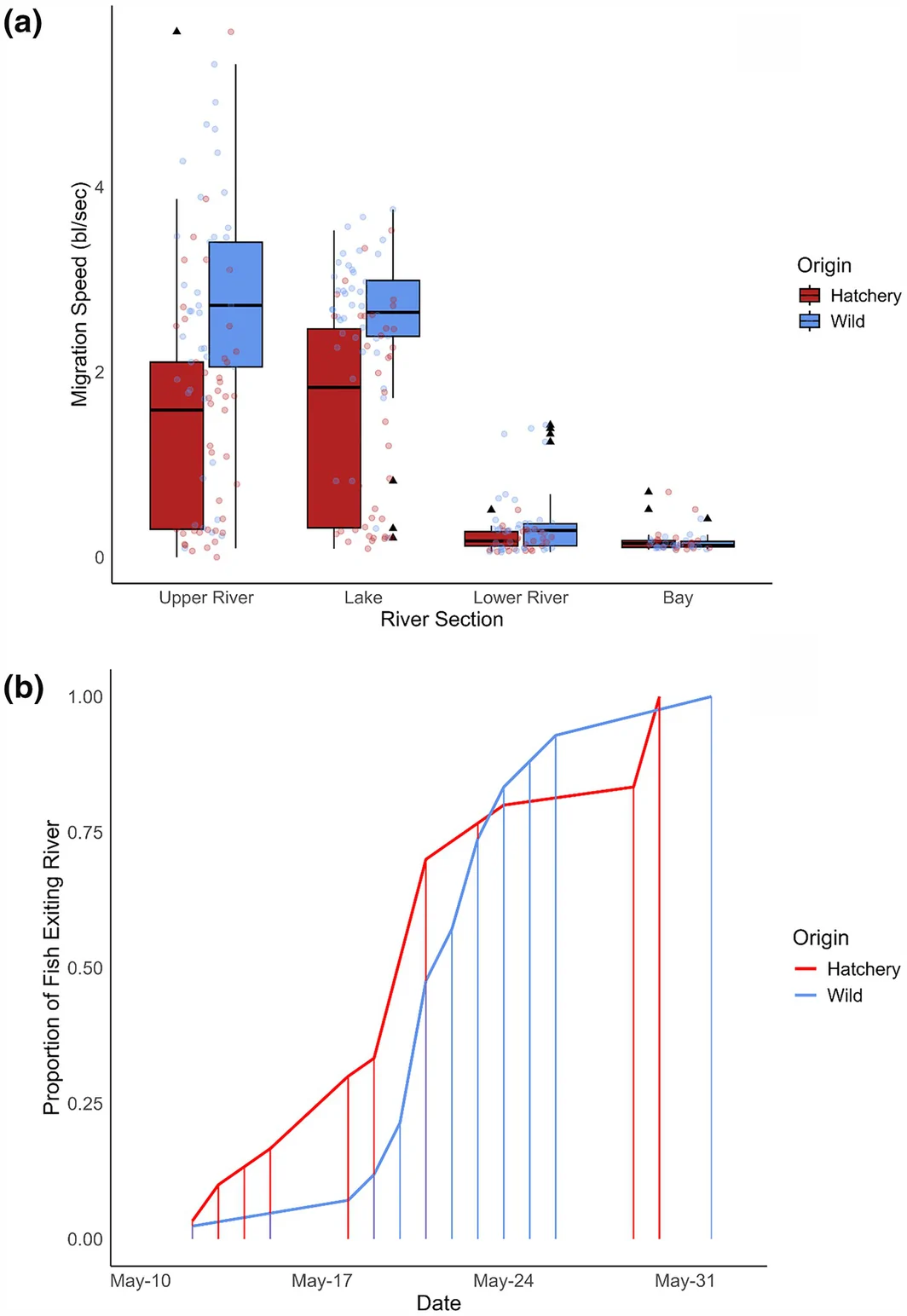
(a) Boxplots of migration speed (body lengths per second) by river section of Atlantic salmon smolts from hatchery and wild origin. The boxes show interquartile range and medians. The black triangles indicate outliers, and points coloured by origin show the raw data. (b) Cumulative proportion and timing of LaHave River exit for hatchery (*n* = 29) and wild (*n* = 41) Atlantic salmon smolt. Each vertical step in the line represents an individual smolt exiting the river, resulting in an increase in the cumulative proportion of fish that have left.

Origin alone had no significant effect on the migration speed (estimate = −0.25 bl/s ± 0.22 SE, *p* = 0.25), but interaction terms showed that wild smolts migrated substantially faster than hatchery smolts in the Upper river (estimate = 0.81 bl/s ± 0.26 SE, *p* = 0.002), Wentzells lake (estimate = 0.75 bl/s ± 0.26 SE, *p* = 0.004) and Lower river (estimate = 0.86 bl/s ± 0.27 SE, *p* = 0.001). Release day and body weight had little effect on migration speed, although smolts released on 3rd May exhibited a moderate increase in the migration speed (estimate = 0.38 bl/s ± 0.14, *p* = 0.009).

Overall, 70 of 98 smolts (29 hatchery, 41 wild) successfully exited the river between 12 May and 1 June. Although wild smolts exhibited faster migration speeds through freshwater sections, this did not translate into an earlier overall river exit, as our analysis of exit timing showed both origins emigrating over a comparable period (Figure 4b). The majority of smolts exited between 18 May and 26 May with little difference between hatchery and wild smolts (Figure 4b). However, reverse movement frequency significantly differed between origins, with hatchery smolts exhibiting more reverse movements than wild smolts in tidally influenced waters during outmigration (GLM: β = −0.61 ± 0.26 SE for wild smolts, *p* = 0.017). Weight and release date were not significant predictors of reverse movement frequency.

### 3.3 Ocean detections

A total of 26 hatchery and 36 wild smolts were detected on five marine acoustic receiver arrays between 13 May and 11 June 2021. Smolts from both origins were detected at similar offshore sites, including the LaHave Islands, Halifax Line and off the coast of Sable Island (Figure 5). Hatchery and wild smolts exhibited similar offshore spatial ranges, as shown by 100% MCPs (Figure 5). Smolts were first detected within the LaHave Islands on 13th May and were consistently detected there until 1st June. Both hatchery (*n* = 11) and wild (*n* = 23) smolts were detected next on the Halifax Line from 21 May to 4 June, including the two that were detected on the southern reaches of the Halifax receiver line. Finally, hatchery (n = 2) and wild smolts (*n* = 4) were both detected approximately 50 km offshore of Sable Island. Two smolts (hatchery *n* = 1, wild *n* = 1) were detected with triggered predation sensors on the Halifax Line and one (hatchery‐origin) near Sable Island. The temperature sensors for the two predated salmon on the Halifax Line detected post‐predation events and recorded temperatures of 18.3°C and 23.9°C, while the Sable Island predation event did not record any temperature data.

**FIGURE 5:**
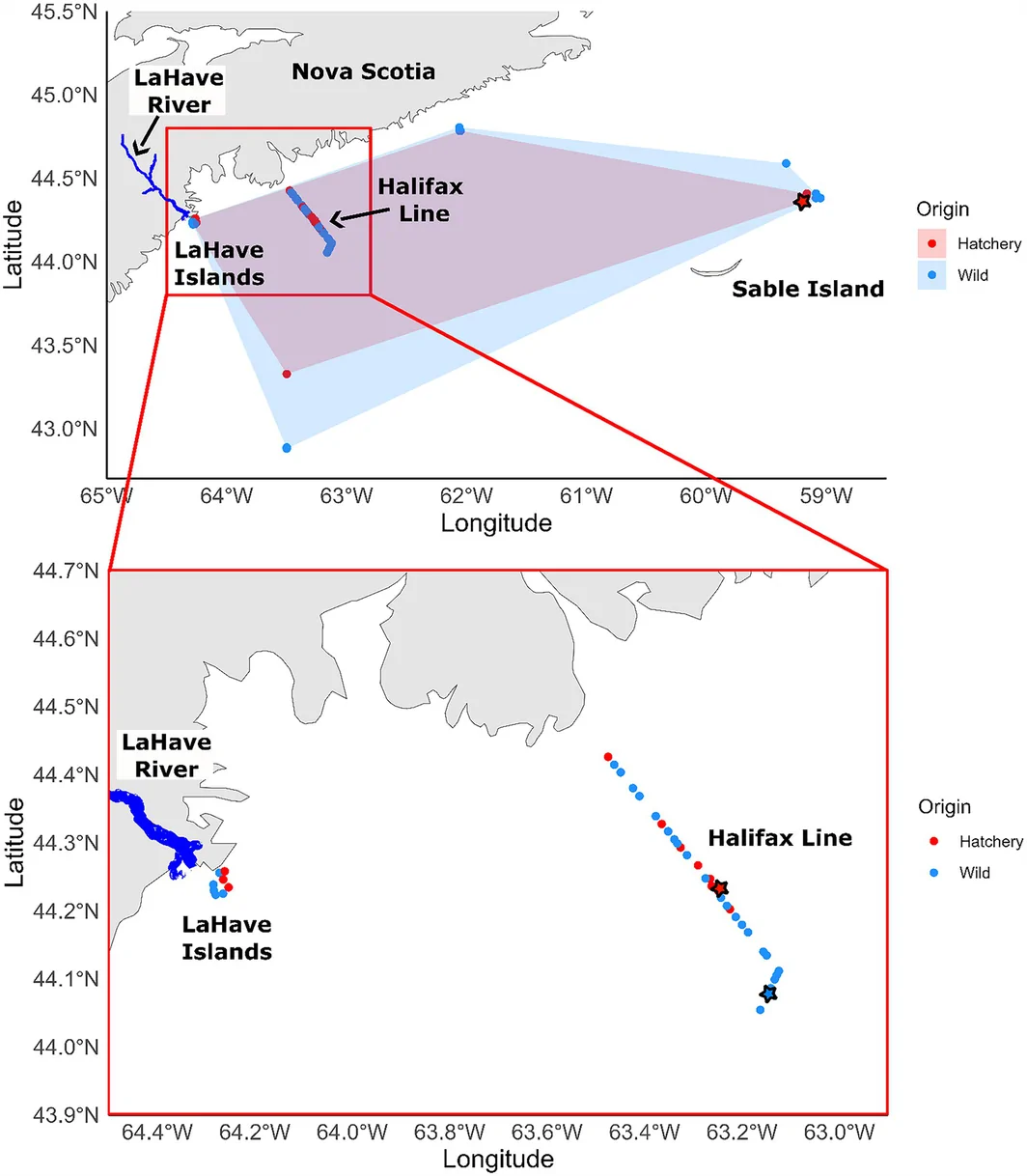
Map showing ocean detections of Atlantic salmon smolts tagged in the LaHave River, Nova Scotia, from hatchery (*n* = 26) and wild (*n* = 36) origins. Individual detections are represented by points, and 100% minimum convex polygons illustrate the spatial range of detections by the origin group. Predation events are represented by the outlined stars (two on the Halifax line, one near Sable Island). Data span from 13 May to 11 June 2021. The inset highlights a closer view of smolt detections near the LaHave Islands and along the Halifax Line.

## 4 DISCUSSION

This study investigated the post‐release behaviour of hatchery‐reared and wild salmon smolts in the LaHave River, Nova Scotia, by comparing survival, predation risk, river migration speed, river exit timing and early ocean migration patterns. Acoustic telemetry revealed important differences between the two origins during outmigration. In comparison to wild smolts, hatchery‐reared individuals exhibited significantly greater mortality, higher predation risk and slower migration speeds across most river sections. Despite this, the timing of river exit (i.e., marine entry) was similar between the successful hatchery and wild smolts, and both were subsequently detected in the same regions during the early stages of the marine migration. These results point to a clear life‐history consequence of hatchery rearing, which has relevance for management concerning the efficacy of current supplementation practices.

Hatchery‐reared smolts experienced significantly lower survival during outmigration compared to wild smolts. In total, hatchery smolts experienced three times higher mortality risk than wild smolts. This high post‐release mortality of hatchery‐reared smolts in the LaHave River system appears to be largely driven by predation, with approximately a third of hatchery‐reared smolts being predated upon after release, compared to the wild smolts <10% predation rate. High mortality in hatchery‐reared smolts due to predation aligns with previous salmonid research, where Kekäläinen et al. (2008) similarly estimated ~29% of stocked Atlantic salmon smolts were predated within 1 week after release. Thorstad et al. (2011) also reported that 45% of released hatchery salmon smolts were lost to predation during early migration. Captively raised smolts are known to exhibit poorer antipredator behaviours, contributing to greater predation rates for hatchery smolts (Olla et al., 1998). These antipredator behavioural deficiencies may include greater surface‐feeding behaviour (Vincent, 1960), a reduced ability to escape predators (Woodward & Strange, 1987), increased movement and quicker return to foraging after exposure to alarm cues (Fleming & Einum, 1997; Jackson & Brown, 2011) or a lack of avoidance in the presence of predators (Johnsson et al., 1996). In natural environments, wild smolts are exposed to predators across multiple years and spaces, where individuals that behave riskily are more likely to be selected against by predation. Survivors learn predator avoidance through experience. Natural exposure contributes to density‐dependent mortality, a process where survival rates decline as population density increases due to intensified competition and predation (Gee et al., 1978; Grant & Kramer, 1990). Under these conditions, only the most fit individuals (i.e., those better able to detect, evade or avoid predators) are likely to survive, shaping behaviour and improving the predator awareness of the surviving smolts (Falkegård et al., 2023; Laundré et al., 2014). In contrast, hatchery‐reared smolts are raised in stable environments with low structural complexity, a lack of predators and routine feedings. As a result, traits important for survival in the wild, such as predator recognition or cautious foraging behaviour, are not developed due to the absence of natural selective pressures. Upon release, their inexperience and lack of conditioning, compounded by the likely stress of captivity handling and the introduction into a novel habitat, may reduce their ability to detect or appropriately respond to predators, increasing vulnerability and ultimately reducing survival.

In this study, the high smolt predation was likely driven by the established populations of invasive Chain pickerel and Smallmouth bass in the LaHave River watershed, which are species recognized as major predators of juvenile salmon (Feener, 2017; Raab et al., 2024; Mensinger et al., 2025). A study conducted on Wentzells Lake found salmon smolts to be the most common prey item in the stomach contents of Chain pickerel (Feener, 2017). Smallmouth bass were also found to have ingested some salmon smolts (Feener, 2017). Furthermore, 85% of invasive predators that had consumed smolts were caught at the top of Wentzells Lake, the same area where most predation occurred in our study. Using tethered Atlantic salmon smolts in a Maine river system, Mensinger et al. (2025) documented that smolts were exclusively preyed upon by non‐native Chain pickerel and Smallmouth bass, with nearly one‐quarter of smolts predicted to be consumed within an hour after a release. This suggests that the invasive species were most likely the primary predators responsible for smolt mortality in our study as well. Other potential aquatic predators are limited, especially with all the predation events occurring in and near Wentzells Lake (e.g., no estuarine or outer bay aquatic predation, suggesting no influential marine predators such as striped bass waiting in the bay), and are therefore unlikely to have played a major role.

Large disparities in freshwater predation between hatchery and wild smolts highlight the need for improved management strategies to enhance in‐river smolt survival. Given the identification of a predation hotspot in Wentzells Lake, a practical management approach for increasing post‐release survival is releasing the hatchery smolts downstream, significantly reducing predation risk and increasing the amount of smolts reaching early marine migration. However, the release location of smolts is also known to influence adult return behaviour. Jonsson et al. (1994) determined that hatchery salmon smolts released near the river mouth (below spawning grounds) hesitated in their spawning migration and ascended later compared to hatchery smolts released in the upper part of the river, which had very similar timing to wild smolts. Therefore, any downstream release strategy must balance reduced juvenile predation risk with potential effects on adult homing and spawning migration success related to early‐life experience in their natal river. Releasing hatchery‐reared smolts further downstream is also contingent on the new release site not being too close to the head of tide, which could impose additional constraints regarding salinity acclimatization. Furthermore, studies have demonstrated that implementing some pre‐release methods for hatchery‐reared smolts could decrease predation events. Vilhunen (2006) trained hatchery‐reared Arctic charr (*Salvelinus alpinus*) to recognize the odour of a predator by repeated exposure. Fish that were conditioned survived longer than predator‐naive controls when later exposed to real predators. Similarly, salmon hatcheries could use predator odours, chemical cues or visual stimuli, such as those from Chain pickerel and Smallmouth bass in the LaHave River, to help smolts develop antipredator responses before release. Finally, morphological characteristics, such as body colouration, can also influence vulnerability to predators. Maynard et al. (2004) found that Chinook salmon reared in tanks with seminatural habitat (such as gravel, structure, dark green colouration and cover) developed more cryptic colouration and had higher post‐release survival compared to those raised in bare tanks, likely due to reduced visibility to predators. Donnelly and Whoriskey (1991) demonstrated that Brook trout (*Salvelinus fontinalis*) fry experienced less avian predation if they were held in tanks with similar colouration as the environment in which they were predated. Thus, colour acclimating the trout changed their morphology, making them more cryptic and less susceptible to avian predation. Overall, to mitigate increased predation risk, hatchery programmes should identify optimal release locations that balance reduced exposure to predation hotspots with the maintenance of a natural homing ability and natal river experience, incorporate predator cue exposure during hatchery rearing to promote the development of antipredator behaviours, and create captive conditions that promote natural, cryptic colouration. Implementing such practices may significantly improve early in‐river survival and increase the number of hatchery‐reared salmon that successfully reach the sea.

Wild smolts migrated significantly faster than hatchery smolts in the Upper river, Wentzells Lake and Lower river. These river section‐specific differences suggest that behavioural responses to different habitats may vary between wild and hatchery smolts, even if their overall time to river exit is comparable. In situ, Gale et al. (2004) reported that hatchery‐reared juvenile steelhead (*Oncorhynchus mykiss*) migrated more slowly than their wild counterparts. *Ex situ*, Pedersen et al. (2008) found that wild salmon smolts performed significantly better than hatchery smolts in a swim flume, swimming 30% faster. Reduced swimming ability, likely due to a lack of exercise and experience with different water flow regimes while reared in captivity, could be a driver in the slower migration speeds we observed in this study's hatchery‐reared smolts.

The reduced migration speed of hatchery smolts may also contribute to the high predation rates observed in Wentzells Lake. Some studies report significant delays and increased predation of hatchery smolts in lakes because they lack navigational cues due to the slower water flow and the habitat offering fewer refuges while containing higher predator densities (Hansen et al., 1984; Kennedy et al., 2018; Schwinn et al., 2019). Disorientation and limited navigational skills after release into a novel wild environment may contribute to the slower migration observed in hatchery smolts, especially in lake habitats with limited flow cues (Olla et al., 1998). In many river systems, smolts must pass through lakes during their migration. Hanssen et al. (2022) found that 60% of tagged Atlantic salmon smolts died in lake habitats, with half of these deaths due to predation. Reduced swimming ability, combined with prolonged residence in a known predator hotspot, likely increases vulnerability to predation, particularly when paired with poorer antipredator behaviours. Despite these challenges, previous studies have observed hatchery smolts match or even exceed wild smolts in outmigration speeds, traversing lakes quickly (up to 15.6 km/day; Bourgeois & O'Connell, 1988; Hansen et al., 1984; Urke et al., 2013). Hyvärinen and Rodewald (2013) demonstrated that hatchery smolts raised in enriched rearing environments (overhead cover, gravel substrate and variable water flow) exhibited faster initial migration speeds than those raised in standard bare tanks. Future management practices could benefit from adopting such enriched rearing strategies. Adding structural complexity and variable flow regimes could provide physical exercise opportunities, improving smolt swimming performance and orientation during outmigration. Enhancing these rearing conditions could increase survival by enabling smolts to evade predators more effectively and move through navigationally challenging habitats more efficiently, thus reducing overall exposure to predators.

Despite consistently slower migration speeds in hatchery smolts, both origins exited the river over the same time period. This convergence likely reflects behavioural and environmental constraints in the lower river and estuary, where smolts respond to shared cues such as flow, temperature, and salinity that synchronize seaward migration (McCormick et al., 1998). However, hatchery smolts exhibited significantly more reverse movements in the lower river, indicating less efficient downstream migration and prolonged estuarine acclimation compared to wild smolts. The greater frequency of reversals by hatchery smolts in the lower river may reflect a reduced ability to navigate transitional habitats for hatchery smolts. Hawkes et al. (2017) observed similar migratory behaviour in hatchery salmon smolts compared to those in regionally non‐stocked rivers, but reversal frequency was higher in hatchery smolts. Additionally, Nilsen et al. (2023) reported that adult hatchery‐reared Atlantic salmon exhibited more reverse movements entering the river upon marine return. Differences in rearing environment between hatchery and wild salmon are known to influence physiological development (Gale et al., 2004; Poole et al., 2003; Strand & Finstad, 2007). For example, warmer or more stable hatchery conditions can accelerate smoltification timing (Strand & Finstad, 2007), potentially leading to earlier physiological development compared to wild smolts. Other studies have reported reduced Na^+^/K^+^‐ATPase activity in hatchery steelhead smolts (Gale et al., 2004), indicating reduced seawater acclimation capacity, which is consistent with observations in this study, where the increased frequency of reverse movements near the head of tide for hatchery smolts was possibly associated with challenges in osmoregulatory adjustment. In either case, both origins appear to have exited the river at the same time despite migration speed differences, suggesting that both groups may ultimately respond to common environmental triggers for marine entry. Outmigration timing is an important determinant of marine smolt survival (McCormick et al., 1998; Rikardsen & Dempson, 2011), and may indicate that hatchery smolts retain some capacity to respond to key environmental cues, despite differences in outmigration movement patterns and potential osmoregulatory capacity.

Hatchery and wild smolt marine detections showed strong spatial and temporal overlap (Figure 5). Both groups followed the expected migration route of first being detected along the southern coast of Nova Scotia (Bowlby et al., 2013), including the Halifax Line, before moving offshore near Sable Island. This route aligns with their expected migration path toward eastern offshore Newfoundland and eventually the Labrador Sea (ESRF, 2023; Reddin & Lear, 1990). Jonsson and Fleming (1993) previously reported that European hatchery‐reared smolts migrated to the same marine feeding areas as wild salmon, which could explain the similar hatchery and wild smolt detections near Sable Island, a biologically rich area within the Eastern Scotian Shelf Canyons known for its high species diversity and ecological importance (DFO, 2014). In addition to origin differences, the marine detection data presented here offer rare empirical evidence of early marine migration routes for the NSSU DU Atlantic salmon populations. The consistent detections along the southern coast of Nova Scotia up to the coast of Sable Island suggest that these areas function as seasonally important migratory habitats during the early marine phase. Identifying these spatially and temporally defined migration corridors is critical for recovery planning. These findings provide valuable context for assessing potential threats from industrial activities and proposed offshore wind developments, ensuring such projects are evaluated in relation to known salmon migration routes and habitat use.

Interestingly, most smolts detected in the marine environment did not have their predation tags triggered, despite the high mortality rates typically associated with early marine migration (Lacroix & Knox, 2005; Thorstad et al., 2012). The lack of marine predation is especially surprising given that Sable Island is home to the largest breeding colony of grey seals (den Heyer & Bowen, 2017), a known predator of Atlantic salmon smolt (Baker et al., 2015; Lenky & Share, 2011) that frequently forage throughout the Scotian Shelf, overlapping with areas where smolts in this study were detected (Breed et al., 2009; Nowak et al., 2020). Despite this, only one of the six smolts detected off Sable Island had its predation tag triggered. While temperature sensors were included on transmitters in this study, unfortunately, this predation event was not accompanied by usable temperature data, limiting our ability to identify the predator species. There are many factors that would cause a tag to fail to report sensor data [environmental interference, signal collision, or reduced transmission quality (e.g., due to tag orientation or ingestion)], resulting in detections where predation was confirmed but temperature data were not successfully logged. Future studies should continue to incorporate temperature sensors with predation tags to distinguish between avian and piscine predators based on post‐ingestion temperature profiles (English et al., 2024; Lennox et al., 2024). Identifying predator type using temperature‐equipped predation tags could clarify whether hatchery and wild smolts face different predation pressures, as behavioural deficiencies may leave hatchery smolts more susceptible to certain predator types, improving understanding of survival outcomes in freshwater and early marine phases.

A limitation of this study is the temporal scope, as it was conducted over a single outmigration season. Smolt survival can vary considerably among years, often in relation to environmental factors such as river conditions (McCormick et al., 1998; Russell et al., 2012). Notably, documented drought conditions on the LaHave River in 2016 and 2020 have been associated with reduced smolt production (Raab et al., 2024), suggesting that flow conditions may strongly influence both survival and outmigration success. Although few studies have quantified the outmigration survival of smolts in this region, available evidence indicates wide interannual variability. For example, Halfyard et al. (2013) reported 100% survival of 30 tagged, wild smolts in the LaHave River during 2010, when the mean discharge in May was 6.81 m^3^s^−1^ (ECCC, 2021). In contrast, survival in this study (2021) was lower during a period of much higher mean May discharge (28.7 m^3^s^−1^; ECCC, 2021), potentially reflecting more favourable conditions in some years relative to others. Taken together, these comparisons suggest that the survival estimates observed in this study are also shaped by yearly‐specific conditions and may not be representative of longer‐term averages. Multiyear studies are ideal to better capture this interannual variability. Additionally, the tracking period was limited to the early phase of marine migration, leaving the subsequent stages of their oceanic movements and survival unknown. As a result, it is unclear whether hatchery and wild smolts differ in their ability to reach key offshore feeding areas and survive later stages of marine migration. Future studies should use tags with extended battery life to track smolt movements beyond the Nova Scotia coast and into later migratory regions such as the Grand Banks and Labrador Sea. Complementary approaches, such as the use of passive integrated transponder (PIT) tags or external tags (e.g., Floy tags), could also help estimate return rates and provide insight into long‐term survival and growth.

Methodological considerations associated with acoustic telemetry should also be acknowledged. Acoustic telemetry is a powerful tool for tracking fish in natural environments that are otherwise difficult to monitor, but it assumes that tagged individuals behave and survive similarly to untagged fish (Matley et al., 2024). Although all smolts in this study were tagged using consistent methods and tag burdens were comparable across individuals, tagging can still influence behaviour and survival in juvenile salmonids (Caputo et al., 2009; Collins et al., 2013; Perry et al., 2013; Wilson et al., 2017). Potential biases may also arise from tag loss or post‐surgical recovery effects (Ammann et al., 2011; Daniels et al., 2021). Additionally, in this study, most mortalities were linked to poikilothermic predators based on temperature‐sensor data, but the remaining unknown fates are less clear. Some smolts may have died between receivers without being consumed, expelled their tags or been transported by an avian or mammalian predator outside the detection area (Lawrence et al., 2023). However, given the high detection efficiencies of the array (83–100%), it is unlikely that either a surviving smolt or a predator carrying a tag passed through unnoticed. The use of predation‐sensor tags reduced the number of potential unknown fates and proved to be a highly effective tool for improving fate classification and highlighting predator hot spots along a migration route. These considerations should be kept in mind when interpreting telemetry‐based survival estimates.

Future studies should integrate both experimental and in situ approaches to better resolve the mechanisms underlying differences between hatchery and wild smolts. Controlled experiments examining how rearing conditions (temperature, flow, structure) influence smoltification physiology and antipredator behaviour would provide a highly valuable baseline in parallel with tracking field studies, linking behavioural and physiological differences to survival outcomes in natural systems. For example, a higher resolution telemetry array within identified predation hotspots, such as Wentzells Lake, could help clarify how habitat use, movement patterns, and predator distribution interact to drive mortality risk. Ultimately, these approaches would provide a more mechanistic understanding of how the rearing environment shapes salmon smolt behaviour, physiology, and survival across life stages.

## 5 CONCLUSION

This study demonstrates that hatchery‐reared Atlantic salmon smolts exhibited significantly reduced survival and increased predation risk during river outmigration compared to their wild counterparts. Hatchery smolts demonstrated significantly slower migration speeds through the upper river and lake sections, and increased reversal frequency at the head of tide. However, river exit timing and early ocean migration patterns were comparable to wild smolts. Despite facing greater mortality in freshwater, hatchery smolts that survived to the ocean showed similar spatial and temporal migration patterns to wild smolts, highlighting that hatchery programmes may support long‐distance migration if early freshwater survival challenges are addressed. These findings highlight the critical need to improve hatchery practices to enhance smolt survival during early freshwater outmigration, ensuring more hatchery smolts successfully transition to the marine environment. Based on our results, we propose the practical recommendation of releasing smolts downstream of predation hotspots, like lakes, which could improve in‐river survival. Integrating controlled experimental studies with in‐situ field investigations is essential to further examine how rearing conditions influence smoltification, physiology and behavioural responses such as predator avoidance and habitat use across freshwater and marine environments. In addition, long‐term tracking efforts, such as monitoring the return of smolts and incorporating tags with extended battery life, should be expanded to better understand marine migration success and determine whether hatchery smolts reach key feeding areas or return to spawn. Moving forward, hatchery rearing and release strategies that incorporate recommended improvements are essential to increasing the return rates of stocked salmon and supporting the long‐term recovery of declining Atlantic salmon populations.

## AUTHOR CONTRIBUTIONS

Natalie C. Koopman: data management & curation, formal analysis, investigation, visualization, writing original draft, writing review & editing. Dave C. Hardie: project conceptualization, fieldwork, data collection, writing review & editing. Robert J. Lennox: formal analysis, writing review & editing, supervision, resources. Morgan L. Piczak: formal analysis, writing review & editing, supervision. Frederick G. Whoriskey: project conceptualization, writing review & editing. Jeffrey A. Hutchings: project conceptualization. Eric B. Brunsdon: fieldwork, data collection, writing review & editing. Glenn T. Crossin: conceptualization, formal analysis, writing review & editing, supervision, resources.

## FUNDING INFORMATION

This work was supported by Fisheries and Oceans Canada through the Marine Threats Program Core Funding. Additional funding was provided through a Natural Sciences and Engineering Research Council of Canada (NSERC) Alliance Grant (ALLRP 586296‐23) and NSERC Discovery Grant (RGPIN‐2021‐02432), both secured by Glenn Crossin.

## CONFLICT OF INTEREST STATEMENT

The authors declare no conflicts of interest.

## Supporting information


**Table S1.** Model selection results for Cormack–Jolly–Seber models evaluating the effects of smolt origin and river section on apparent survival (ϕ) and detection probability (p). Models are ranked by Akaike's information criterion corrected (AICc) for small sample size, with K representing the number of parameters in the model.
**Table S2.** Detection probability (p) of smolts at each acoustic receiver from the Cormack–Jolly–Seber (CJS) model (p ~ receiver). Detection probability represents the probability that a fish was detected, assuming it was alive and present at the receiver. Estimates are shown with standard errors and 95% confidence intervals.
**Table S3.** Cumulative apparent survival (ϕ) of hatchery and wild smolts through each river section (upper river, lake, lower river, bay). Estimates reflect survival from release to the end of each river section. Standard errors and 95% confidence limits (LCL, UCL) were derived from the Cormack–Jolly–Seber addition model.
**Table S4.** Fixed‐effect parameter estimates from the generalized linear mixed‐effects model (Gamma distribution, log link) describing variation in migration speed (body lengths per second).
**Figure S1.** Acoustic transmitter sensor time series of four tagged smolts. Individuals in panel (a) show a predation event, indicated by the activation and increase of the predation sensor value (P). Individuals in panel (b) show the predation sensor did not activate, indicating no predation event occurred. The bottom ‘T’ box for each smolt's time series denotes the temperature sensor.

## Data Availability

The data that support the findings of this study are available from the corresponding author upon reasonable request.
