# Supplementary material for: Tracking hatchery and wild Atlantic salmon (Salmo salar) smolt during river exit and early marine migration to assess survival, predation risk and migration speed
Source: J Fish Biol. 2026 Jul 30;109(4):2231–47. doi: 10.1111/jfb.70599 (PMC13675484; doi:10.1111/jfb.70599)
Supplement: Supplementary file 1 [file JFB-109-2231-s001.docx]

**Supplementary material**

**Tables**

**Table S1:** Model selection results for Cormack-Jolly-Seber models evaluating the effects of smolt origin and river section on apparent survival (ϕ) and detection probability (p). Models are ranked by Akaike’s information Criterion corrected for small sample size (AICc) with K representing the number of parameters in the model.

| **Model** | **K** | **AICc** | **ΔAICc** | **Model weight** |
| --- | --- | --- | --- | --- |
| ϕ(origin +  river section), p(river section) | 34 | 559.53 | 0.00 | 0.95939 |
| ϕ(river section), p(river section) | 33 | 565.85 | 6.33 | 0.04058 |
| ϕ(origin ×  river section), p(river section) | 49 | 581.30 | 21.78 | 0.00002 |
| ϕ(origin),  p(river section) | 19 | 684.89 | 125.36 | 0 |

**Table S2.** Detection probability (p) of smolts at each acoustic receiver from the Cormack-Jolly-Seber (CJS) model (p~receiver). Detection probability represents the probability a fish was detected assuming it was alive and present at the receiver. Estimates are shown with standard errors and 95% confidence limits intervals.

| **River section** | **Receiver** | **Estimated**  **p** | **Standard error** | **LCL** | **UCL** |
| --- | --- | --- | --- | --- | --- |
| Release | 1 | 0.0510204 | 0.022 | 0.021 | 0.117 |
| Upper River | 2 | 1.0 | <0.010 | 0.999 | 1.0 |
| Upper River | 3 | 0.989 | 0.010 | 0.930 | 0.999 |
| Upper River | 4 | 1 | <0.010 | 0.999 | 1.0 |
| Lake | 5 | 0.989 | 0.010 | 0.927 | 0.998 |
| Lake | 6 | 0.989 | 0.010 | 0.927 | 0.998 |
| Lake | 7 | 1.0 | <0.010 | 0.999 | 1.0 |
| Lower River | 8 | 0.908 | 0.033 | 0.819 | 0.955 |
| Lower River | 9 | 0.986 | 0.013 | 0.911 | 0.998 |
| Lower River | 10 | 1.0 | <0.010 | 0.999 | 1.0 |
| Lower River | 11 | 1.0 | <0.010 | 0.999 | 1.0 |
| Lower River | 12 | 1.0 | <0.010 | 0.999 | 1.0 |
| Lower River | 13 | 0.917 | 0.033 | 0.827 | 0.962 |
| Outer Bay | 14 | 0.944 | 0.027 | 0.861 | 0.979 |
| Outer Bay | 15 | 1.0 | <0.010 | 0.999 | 1.0 |
| Outer Bay | 16 | 0.625 | 0.057 | 0.508 | 0.729 |

**Table S3.** Cumulative apparent survival (ϕ) of hatchery and wild smolts through each river section (upper river, lake, lower river, bay). Estimates reflect survival from release to the end of each river section. Standard errors and 95% confidence limits (LCL, UCL) were derived from the Cormack-Jolly-Seber addition model.

| **River section** | **Origin** | **Cumulative survival** | **Cumulative standard error** | **LCL** | **UCL** |
| --- | --- | --- | --- | --- | --- |
| Upper River | Hatchery | 0.984 | 0.016 | 0.895 | 0.998 |
| Upper River | Wild | 0.995 | <0.010 | 0.964 | 0.999 |
| Lake | Hatchery | 0.890 | 0.039 | 0.785 | 0.947 |
| Lake | Wild | 0.966 | 0.015 | 0.920 | 0.986 |
| Lower River | Hatchery | 0.605 | 0.063 | 0.476 | 0.720 |
| Lower River | Wild | 0.859 | 0.034 | 0.779 | 0.914 |
| Bay | Hatchery | 0.605 | 0.063 | 0.476 | 0.720 |
| Bay | Wild | 0.856 | 0.034 | 0.779 | 0.914 |

**Table S4.** Fixed-effect parameter estimates from the Generalized Linear Mixed-effects Model (Gamma distribution, log link) describing variation in migration speed (body lengths per second).

| **Predictor** | **Estimate** | **SE** | **z** | **p** |
| --- | --- | --- | --- | --- |
| **Intercept** | -1.7336 | 0.2555 | -6.786 | <0.001 |
| **River section (ref=Bay)** |  |  |  |  |
| Upper River | 2.0701 | 0.1907 | 10.853 | <0.001 |
| Lake | 2.1236 | 0.1932 | 10.992 | <0.001 |
| Lower River | 0.0201 | 0.2033 | 0.099 | 0.921 |
| **Origin (ref = hatchery)** |  |  |  |  |
| Wild | -0.2509 | 0.2164 | -1.159 | 0.246 |
| **Body weight** | -1.1544 | 1.6263 | -0.710 | 0.478 |
| **Release day (ref=April 29)** |  |  |  |  |
| May 1 | 0.1571 | 0.1527 | 1.029 | 0.304 |
| May 3 | 0.3772 | 0.14412 | 2.617 | 0.009 |
| May 5 | -0.0694 | 0.1493 | -0.465 | 0.642 |
| May 7 | 0.1675 | 0.1531 | 1.094 | 0.274 |
| May 9 | 0.2099 | 0.1795 | 1.170 | 0.242 |
| **Interactions (River section x Origin** |  |  |  |  |
| Upper River x Wild | 0.8105 | 0.2582 | 3.139 | 0.002 |
| Lake x Wild | 0.7535 | 0.2608 | 2.889 | 0.004 |
| Lower River x Wild | 0.8640 | 0.2698 | 3.203 | 0.001 |

**Figures**

**Figure S1.** Acoustic transmitter sensor time series of four tagged smolts. Individuals in panel a) show a predation event, indicated by the activation and increase of the predation sensor value (P). Individuals in panel b) show the predation sensor did not activate, indicating no predation event occurred. The bottom “T” box for each smolts time series denotes the temperature sensor.
